# Retinal adaptive optics imaging with a pyramid wavefront sensor

**DOI:** 10.1364/BOE.438915

**Published:** 2021-09-02

**Authors:** Elisabeth Brunner, Julia Shatokhina, Muhammad Faizan Shirazi, Wolfgang Drexler, Rainer Leitgeb, Andreas Pollreisz, Christoph K. Hitzenberger, Ronny Ramlau, Michael Pircher

**Affiliations:** 1 Center for Medical Physics and Biomedical Engineering, Medical University of Vienna, Waehringer Guertel 18-20, A-1090 Vienna, Austria; 2 Johann Radon Institute for Computational and Applied Mathematics, Altenbergerstrasse 69, A-4040 Linz, Austria; 3 Department of Ophthalmology and Optometry, Medical University of Vienna, Waehringer Guertel 18-20, A-1090 Vienna, Austria; 4 Johannes Kepler University Linz, Industrial Mathematics Institute, Altenbergerstrasse 69, A-4040 Linz, Austria

## Abstract

The pyramid wavefront sensor (P-WFS) has replaced the Shack-Hartmann (SH-) WFS as the sensor of choice for high-performance adaptive optics (AO) systems in astronomy. Many advantages of the P-WFS, such as its adjustable pupil sampling and superior sensitivity, are potentially of great benefit for AO-supported imaging in ophthalmology as well. However, so far no high quality ophthalmic AO imaging was achieved using this novel sensor. Usually, a P-WFS requires modulation and high precision optics that lead to high complexity and costs of the sensor. These factors limit the competitiveness of the P-WFS with respect to other WFS devices for AO correction in visual science. Here, we present a cost-effective realization of AO correction with a non-modulated P-WFS based on standard components and apply this technique to human retinal *in vivo* imaging using optical coherence tomography (OCT). P-WFS based high quality AO imaging was successfully performed in 5 healthy subjects and smallest retinal cells such as central foveal cone photoreceptors are visualized. The robustness and versatility of the sensor is demonstrated in the model eye under various conditions and *in vivo* by high-resolution imaging of other structures in the retina using standard and extended fields of view. As a quality benchmark, the performance of conventional SH-WFS based AO was used and successfully met. This work may trigger a paradigm shift with respect to the wavefront sensor of choice for AO in ophthalmic imaging.

## 1. Introduction

With the rise of adaptive optics (AO), the potential of high resolution optical imaging has been unlocked for several applications including astronomy [1], microscopy [2] and ophthalmology [3]. AO enables imaging in or close to the diffraction limit by measuring and correcting wavefront aberrations introduced by propagation of the light through inhomogeneous media or rough interfaces. In ophthalmology imperfections of the eye degrade the retinal imaging quality and AO is used in combination with different imaging modalities such as fundus photography [3], scanning laser ophthalmoscopy (SLO) [4], or optical coherence tomography (OCT) [5, 6] for an *in vivo* visualization of cellular structures. With the resulting imaging capabilities of these instruments, various cell types such as cone photoreceptors [4, 7], rod photoreceptors [8, 9], retinal pigment epithelium (RPE) cells [10, 11], ganglion cells [12] and more have been identified in the retina from data captured *in vivo*. These works have revolutionized the imaging options of the living retina, and first commercialization of AO supported instruments for improved diagnostics and treatment control have been realized.

In classical AO, wavefront aberrations are measured with a wavefront sensor (WFS) and then compensated by a deformable mirror (DM) by adapting its shape. One option of a WFS is the pyramid wavefront sensor (P-WFS) [13] that has originally been introduced in astronomical AO. The sensor’s main component is a multi-facet glass pyramid with a large vertex angle, where the tip of the pyramid is placed in the system’s focal plane and the light is split into as many parts as there are facets. For each part, an image is formed on the detector which is optically conjugated to the pupil plane of the system. In the presence of wavefront aberrations, the images corresponding to each facet show differing intensity distributions. The resulting pupil images are processed as such or combined into two slope-like data vectors [14]. The sensor data is then used to either reconstruct a wavefront estimate [15] which is subsequently mapped on a DM or to directly compute the DM actuator commands. Since the aberration range for which the P-WFS has a linear response is small, a modulation is in general applied by either oscillating the beam over the pyramid [16] or by oscillating the pyramid itself [13]. Increasing the radius of the modulation leads to a larger dynamic range of the P-WFS with a trade off in the sensitivity of the sensor [14, 17]. In closed-loop AO correction, the modulation is therefore typically dynamic with a larger radius applied at the start, and a smaller radius once the largest bulk of the aberrations has been corrected. The P-WFS was also successfully implemented without modulation by placing a light diffusing plate in an intermediate pupil plane [18]. With adjustable diffusing angles, a blur effect equivalent to that obtained by a dynamic modulation can be obtained without using any moving part.

AO systems for retinal imaging commonly use the well-known Shack-Hartmann wavefront sensor (SH-WFS) [19] which samples the wavefront by the means of an array of lenslets located in a plane conjugated to the pupil of the eye [3, 4, 7–12]. Non-zero wavefront aberrations cause displacements of the created focal spots at the area detector which provide a measure of the mean wavefront gradients over the corresponding lenslets. While the SH-WFS benefits from the large range in aberration strength over which it has a linear response, the sensor is constrained to a fixed sampling of the wavefront defined by the number of lenslets. Further, the center of gravity computation required for localization of the focal spots is a non-obvious task that is sensitive to intensity variations across the lenslets which for example occur at the borders of the pupil [20, 21]. In theoretical analysis [22, 23], simulations [24], bench studies [16, 25] and first on-sky results [26, 27] from the astronomical field, the P-WFS shows better sensitivity than the SH-WFS in closed-loop application, an advantage that is enhanced in low light scenarios and for partly corrected wavefronts. These aspects are directly transferable to ophthalmic AO. A further advantage of the P-WFS is that the pupil sampling can be easily adjusted [13], to match for example the degrees of freedom of the DM which could be advantageous in woofer-tweeter AO systems [28]. While most AO systems currently in operation at the major ground-based telescopes are based on the SH-WFS, the P-WFS is the sensor of choice for a number of high performance AO systems that will be installed at future extremely large telescopes [29, 30]. In the field of visual science, application of the P-WFS has been proposed in the context of ocular aberrometry [31, 32] and AO aberration correction [33, 34]. With respect to the former, clinical evaluations of the technology have been published and several ocular pyramidal aberrometers are available on the market. In the first works regarding ophthalmic AO aberration correction, closed-loop correction was achieved with a P-WFS [33, 34], but no high quality retinal imaging that would allow for an assessment of the AO correction quality could be provided.

In this work, we present the design and implementation of a low-cost 4-sided P-WFS for ophthalmic AO imaging. Neither the use of modulation nor a diffusive element are necessary for AO loop convergence which largely simplifies the design of the sensor and increases its applicability for the field of ophthalmology. No high precision components were used but a standard glass pyramid was implemented. To the best of our knowledge, for the first time high performance *in vivo* AO imaging of the retina using a P-WFS was achieved. The presented P-WFS provides state-of-the-art AO retinal imaging capabilities, visualizing cone photoreceptors in the most central point of the fovea, rod photoreceptors, RPE and ganglion cells. The AO retinal imaging quality obtained with the P-WFS was successfully tested in 5 healthy subjects and for various retinal imaging scenarios to demonstrate the robustness, flexibility and applicability of the sensor. The performance of the P-WFS was assessed in parallel to a SH-WFS [11, 35, 36] using AO-OCT by visualizing *in vivo* smallest cone photoreceptor cells that are located in the central fovea of healthy volunteers. The AO loop is driven by either sensor for comparison using comparable pupil sampling and standard calibration procedures for both sensors. In the presented configurations, AO-OCT using the P-WFS achieves equivalent or even better performance than using the SH-WFS, motivating further in depth comparison of the two sensors and a renewed consideration of the P-WFS as an attractive alternative for sensor-based AO correction in visual science.

## 2. Methods

We have chosen OCT as imaging modality for the *in vivo* demonstration of AO wavefront correction with a non-modulated P-WFS for retinal imaging, but the concept can be directly translated to SLO imaging.

### 2.1 AO-OCT system, assembly and alignment of the P-WFS

**Fig. 1.**
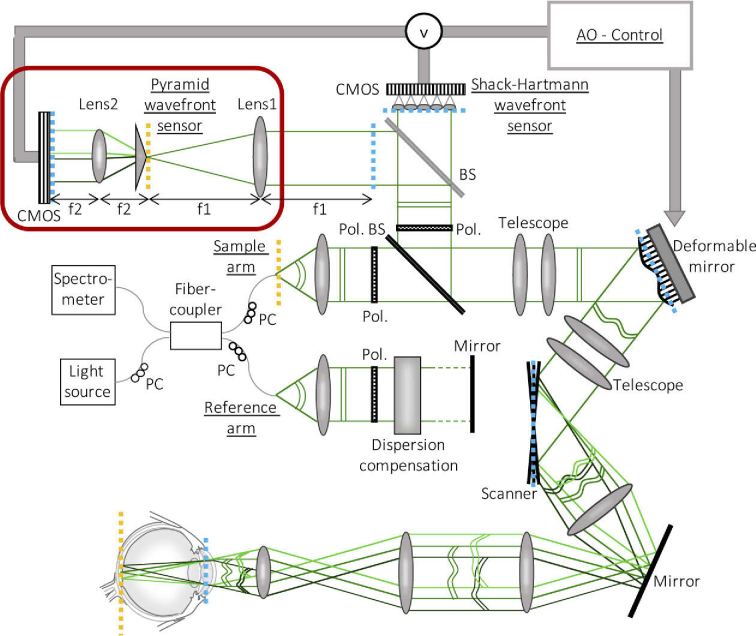
Scheme of the AO-OCT system with a non-modulated P-WFS and a SH-WFS. For simplification only one galvanometer scanner and only 2 of the 4 beams created by the P-WFS are drawn. PC: polarization controller, Pol.: Polarizer, (Pol.) BS: (polarizing) beam splitter. The dashed blue and yellow lines mark pupil and focal planes of the system, respectively

The used setup is a lens based spectral domain AO-OCT system as described earlier [11]. In this study, wavefront aberrations introduced by the eye and the system are measured either with the newly implemented non-modulated P-WFS or with a customized SH-WFS [11, 35, 36] using a 22 × 22 lenslet array (see **Fig. 1**). Wavefront aberration compensation is applied with a DM driven by 69 actuators from the company ALPAO (Montbonnot, France). Wavefront sensing is performed using the light back-scattered from the retina that is in an orthogonal polarization state compared to the linear polarization state incident to the eye [11]. OCT imaging is done with a spectrometer that consists of a collimator, a diffraction grating, a focusing lens, and a line scan camera (spL4096-140 km, Basler, Ahrensburg, Germany). The system supports retinal scanning angles of up to 4° x 4° and records volume data of the retina with a 250 kHz A-scan (depth profile) rate. The imaging light has a center wavelength of 840 nm with 50 nm bandwidth which yields a theoretical axial resolution of 4.5 µm in tissue.

The additional components that are required to implement AO with a non-modulated P-WFS are depicted in **Fig. 1**. In the wavefront sensing path of the system, a non-polarizing 50/50 cube beam splitter is introduced directly in front of the SH-WFS in order to direct half of the light to the P-WFS. With this configuration, wavefront aberrations can be measured with either sensor and potential non-common-path aberrations introduced by the different light pathways to the SH-WFS and the P-WFS, respectively, are reduced to a minimum. The non-modulated P-WFS is assembled from a four faceted glass pyramid with a large vertex angle, two achromatic lenses and a CMOS detector. The first lens (Lens1, f1 = 100 mm) is placed at a distance corresponding to one focal length from a conjugated pupil plane of the system (cf. blue dashed line in the wavefront sensing arm in **Fig. 1**). The sensing light is focused at the tip of a glass pyramid (custom made) which has a vertex angle of 160° and the dimensions 25.4 mm × 25.4 mm × 7.2 mm. Unlike in P-WFS based astronomical AO, no high precision component was used, but the pyramid was produced with standard optical manufacturing processes resulting in the following accuracies: The roof has a width of ∼120 µm and the edges of ∼15 µm. The cost of the pyramid is 500 € per unit. The vertex of the pyramid is positioned in the back focal plane of the first lens (cf. yellow dashed line in **Fig. 1**). Due to the double-pass configuration of the imaging and detection light, the pyramid vertex is optically conjugated to the fiber tip emitting the imaging light and to the focal spot created on the retina. The theoretical width of the point spread function at the pyramid tip is ∼4.4 µm, calculated at the sensing wavelength of 840 nm. The pyramid splits the field into four parts and introduces four different tilts, angularly separating the four beams. A second lens (Lens2, f2 = 30 mm) is used to conjugate the plane of the detector, a CMOS camera (Photonfocus QR1 -D2048 × 1088-384-G2-8), to the pupil. The diameter of the four pupil images is ∼2.2 mm and each contains up to ∼400 samples across the diameter according to the amount of digital pixel binning that is applied. A custom graphic user interface written under Labview (National Instruments, Austin, USA) and MATLAB (The Mathworks, Inc., Natick, USA) processes the pupil images.

During preliminary tests of the implemented non-modulated P-WSF, we observed that very exact alignment of the P-WFS components was crucial to obtain the AO correction performance required for the visualization of smallest retinal cells such as central foveal cone photoreceptors. Both lenses and the detector were therefore mounted on mechanical stages that allow for lateral and axial adjustments in the micrometer range. The pyramid is attached to the stage of the reimaging lens Lens2 by means of a cage system in which the pyramid can be manually rotated and moved along the optical axis independently from the lens. The alignment was performed with a flat mirror inserted between the first telescope and the DM to minimize the effect of systems aberrations on the alignment procedure. The mirror was adjusted such that the light was coupled back into the single mode fiber (which ensures that the light beam is back-reflected in itself). In the first step of the P-WFS assembly, the CMOS camera was placed in the WFS arm orthogonally to the collimated beam at a distance behind the pupil plane in the P-WFS branch which corresponds to the 4f configuration depicted in **Fig. 1**. The center pixel of the detector was laterally aligned to the peak of the Gaussian beam profile. Subsequently, Lens1 was mounted at distance f1 behind the pupil plane and adjusted laterally to place the peak of the diverging beam profile on the center pixel of the detector. Then, Lens2 was added at distance f1+f2 behind the first lens and laterally aligned in the same manner. We verified that the peak of the beam profile did not change position on the detector when Lens2 is axially moved, which confirmed that the lenses were placed orthogonally to the beam. In order to guarantee exact axial alignment of the reimaging lens, the beam was diverted by means of a flat mirror inserted between Lens2 and the detector. The axial position of Lens2 was then fine-tuned until collimation of the diverted beam could be observed over a distance of 5 m. Before the pyramid could be introduced, the axial position of the camera had to be optimized in order to achieve conjugation of the detector to the pupil plane of the system. For this purpose, Lens1 was removed from its mount and the axial position of the camera was adjusted such that the diameter of the focal spot created in the detector by Lens2 was minimized. The pyramid was then mounted into the cage system which is laterally centered with Lens2, while Lens1 remained removed from the assembly. By minimizing the diameter of the four focal spots appearing at the detector, the axial position of the pyramid was determined and rotational alignment was achieved by placing the focal spots in a square with the sides parallel to the axes of the detector. For the fine tuning of the axial and lateral alignment of the pyramid, Lens1 was reinstalled in the system and a model eye (which consists of a lens and a scattering surface) placed at the eye’s pupil location was used as imaging object (the flat mirror between the first telescope and the DM was removed). During detector exposure the galvanometer scanners were turned on and the imaging beam was moving over the surface with a small scanning angle. Since the P-WFS was implemented as an add-on to an existing complete system, we chose to correct for system aberrations during alignment to avoid the possibility of a negative influence of the system aberrations on the final AO performance of the P-WFS. This corrective shape of the DM was computed with the SH-WFS, but it is also possible to correct for systems aberrations with a sensor-less approach [37, 38]. It is important to mention that the P-WFS could also be aligned in a separate setup without system aberrations using a defined point light source and then be added to the system (as was done for the SH-WFS). The stage which holds both Lens2 and the pyramid was now moved laterally until the intensity power was equally distributed among the four pupil images produced by the P-WFS and axially until the intensity was distributed as uniformly as possible within the pupil images.

**Fig. 2.**
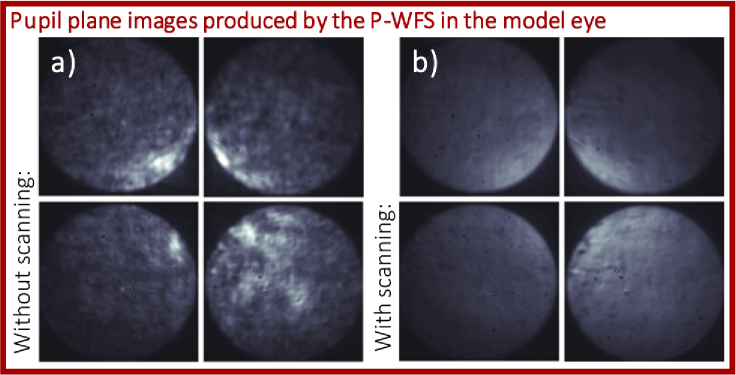
Cut outs of the four pupil plane images produced by the P-WFS without scanning in a) and with scanning in b). The images were obtained in a model eye under the presence of system aberrations and a slight defocus (overall wavefront error RMS 270 nm, measured with the SH-WFS).

One key feature of the system has proven crucial for the successful implementation of the non-modulated P-WFS for ophthalmic AO: Instead of using a second light source for wavefront sensing, part of the imaging light returning from the retina is used to illuminate both WFSs. This configuration has already been highly beneficial for our SH-WFS based AO [11, 35, 36]. Inhomogeneities in the WFS data that are introduced by the sample structure are averaged out through the fast scanning of the beam over the retina, and reflexes from the lens interfaces and the cornea can be greatly attenuated. Residual reflections from the lens interfaces are removed by polarization optics as only light in a crossed polarization state (in respect to the linear state incident to the eye) is directed to the wavefront sensors [11]. Another aspect of using the imaging light for wavefront sensing is that the size of the focal spot on the retina reduces with the AO correction which improves the wavefront sensing performance. The assets of this technique do all translate to the P-WFS and most importantly allow to bypass the need for modulation, i.e., applying oscillation of the sensor or the beam, which greatly reduces complexity and costs of the sensor. In **Fig. 2**, (pupil) images are shown that were obtained from the P-WFS under the presence of residual system aberrations and slight defocus while using a model eye as imaging object. The images in **Fig. 2(a)** were acquired without scanning of the imaging beam across the scattering surface. High frequency intensity variations introduced by the object structure can be clearly observed that degrade the performance of the P-WFS (and are usually removed by sensor modulation). In the presented setup, these are eliminated as soon as the galvanometer scanners are turned on (cf. **Fig. 2(b)**), because light returning from different locations of the object reaches the P-WFS during the exposure time of the sensor and as such the influence of the object structure is averaged out. This is an inherent feature of all scanning ophthalmic imaging modalities and there is no need for an additional modulation implementation. Since the wavefront sensing light is part of the imaging beam, the placement of the focal point on the pyramid tip is hereby guaranteed (in scattering objects) even for large scanning angles due to the double pass configuration of the illumination and detection light (see **Fig. 1**) where the tip of the pyramid lies in a conjugated plane to the tip of the fiber emitting the imaging light. The quality of the P-WFS data greatly improves once the scanners are moving and solely aberration induced intensity variations remain in the pupil images. It should be mentioned that an implementation of the non-modulated P-WFS with a second light source for wavefront sensing is likely to be successful as well, as long as the WFS light traverses the imaging optics including the DM and the scanners.

### 2.2 P-WFS data pipeline and calibration

**Fig. 3.**
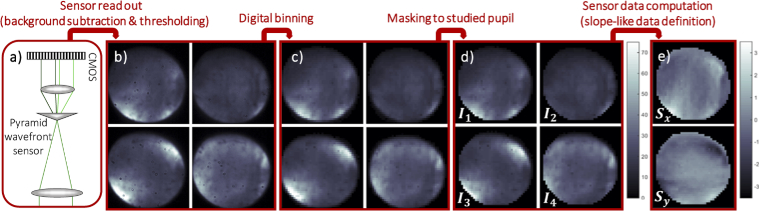
Data pipeline implemented for the non-modulated P-WFS depicted in a): Cut outs of the four pupil plane images obtained from the P-WFS after background subtraction and intensity thresholding are shown in b), followed by the output of the digital binning routine (set amount: 10 × 10 pixels) in c). Only the illuminated pixels within the studied pupil in d) are used according to the pupil image numbering for the computation of the two data maps in e) via the standard approach of slope-like P-WFS data definition. The data was obtained *in vivo*.

The P-WFS can be used for closed-loop AO correction with standard calibration techniques using slope like P-WFS data definition. In **Fig. 3**, the customized P-WFS data pipeline is illustrated with pupil images obtained *in vivo* for volunteer V1 (see the first part of **Supplement 1** for subject characteristics). The sensor data is computed from the four reimaged pupils recorded on the CMOS camera (cf. **Fig. 3(a)**).

The software reads out a pixel area of 1080 × 1080 pixels with each pupil image containing ∼410 pixels in diameter. After subtraction of a pre-recorded background illumination frame and application of an intensity threshold (cf. **Fig. 3(b)**), digital binning is applied via pixel value averaging (cf. **Fig. 3(c)**). For the binning of pixel groups at the borders of the pupil which are partly located inside and partly outside of the threshold mask, only pixels within the threshold mask are averaged in order to avoid introduction of errors at the edges of the pupil. For the presented AO data, several amounts of pixel binning were applied resulting in between 13 and 391 binned pixels across the pupil diameter. During *in vivo* AO imaging, the eye pupil may change in size or move laterally. The effective pupil in each AO correction step is therefore defined as intersection between the studied pupil (pre-defined in the calibration procedure) and the pupil defined by the intensity thresholding. The resulting four slightly different pupil images (cf. **Fig. 3(d)**) can be either used directly (full-frame approach), or are combined into two slope-like data maps. Thorough theoretical analyses of the forward model of the pyramid sensor [39, 40] provide explicit mathematical expressions connecting the sensor data with the corresponding incoming wavefront. In this work the standard slope-like P-WFS data definition is applied and the P-WFS “slopes” $S_{x}$ and $S_{y}\;$ are computed at each point (x,y) of the effective pupil using the following formulas [14]:

(1) $$S_{x}(x,y)=\frac{1}{I_{0}}[\left(I_{1}(x,y)+I_{4}(x,y))-(I_{2}(x,y)+I_{3}(x,y)\right)],$$

(2) $$S_{y}(x,y)=\frac{1}{I_{0}}[\left(I_{1}(x,y)+I_{2}(x,y))-(I_{3}(x,y)+I_{4}(x,y)\right)],$$

where $I_{i}(x,y)$ is the intensity at point (x,y) in pupil image *i* and the pupil images at the CMOS camera are numbered according to **Fig. 3(d)**. A normalization is applied by $I_{0}$ which is the mean intensity in the four pupil images where only the effective pupil is considered. For small aberrations, the normalized intensity differences in **Eqs. (1)** and **(2)** are proportional to the vertical and horizontal wavefront gradients. The P-WFS data points within the studied pupil that are not illuminated are set to zero. The resulting P-WFS data maps contain also information about the observed tip and tilt modes which do not have to be corrected due to the double-pass configuration of the imaging and detection light. Tip and tilt can be filtered from the P-WFS data by subtracting, respectively, the mean value of $S_{x}(x,y)$ and $S_{y}(x,y)$ computed over the effective pupil from the slope maps, providing the final P-WFS data maps shown in **Fig. 3(e)**.

**Fig. 4.**
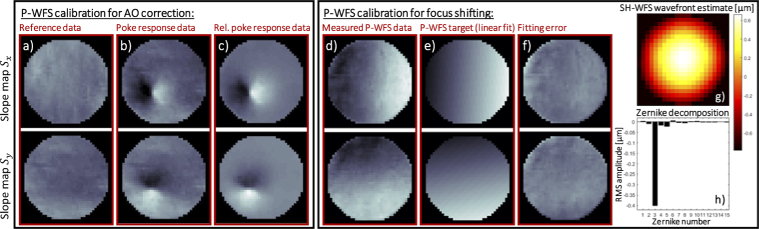
P-WFS calibration data for computation of the sensor response to 20% stroke at actuator Nr. 25 (left box) and target computation for closed-loop focus shifting with the P-WFS (right box). The reference P-WFS data maps are shown in a) next to the absolute P-WFS response maps in b) and the relative P-WFS response maps in c). The P-WFS data maps measured for a defocus wavefront of 0.4 µm root mean square (RMS) amplitude are shown in d), followed by the target data maps computed through a linear polynomial fit of the measured data in e) and the polynomial fitting error in f). The defocus wavefront was measured with the SH-WFS, where g) shows the wavefront estimate and h) the corresponding Zernike decomposition (including the first 15 modes according to the Noll definition [42], except the piston mode).

The DM actuator commands required for compensating the ocular aberrations are calculated directly from the P-WFS data through a zonal control approach [15]. The system was calibrated in the model eye for closed-loop operation while the galvanometer scanners were moving the beam over the object with a small scanning angle of 0.5°. P-WFS data maps were acquired for the poking action of each DM actuator. The pseudo-inverse of the resulting interaction matrix was obtained via a truncated singular value decomposition [41] and used as AO control matrix for computing the closed-loop commands of the DM actuators. In the presented calibration procedure, we assume a linear relationship between actuator commands and sensor responses. As mentioned in the introduction of the main manuscript, the P-WFS shows linear behavior for only very small wavefront aberrations and generally dynamic modulation is applied to increase the dynamic range of the sensor [13, 14, 17]. In this work, AO correction with the P-WFS is achieved without beam modulation. It has to be subject of future investigations, to which extent the averaging applied by the scanning of the imaging beam across the retina increases the linear range of the P-WFS similar as it has been shown for beam modulation. In order to account for potential non-linearity, we performed the calibration close to zero aberrations which minimizes the error in linearization of a nonlinear process. For this purpose, an actuator command vector correcting for system aberrations was added to the bias command vector of the DM. This pre-correction was computed with the SH-WFS of the system, but could alternatively be obtained through a sensor-less approach. At a later stage of this work, we performed the P-WFS calibration without pre-correction of the system aberrations and found that the resulting *in vivo* AO imaging performance was comparable to the quality achieved with a pre-correction in the calibration step. This preliminary result suggests that the P-WFS calibration is robust to small system aberrations. The studied pupil is now defined based on the recorded pupil images after intensity thresholding and the desired amount of digital binning. It has proven beneficial for the AO performance achieved with this P-WFS implementation to reduce the resulting mask by a small number of outer circles of binned pixels. Before applying the actuator pokes, a reference frame was recorded with the P-WFS and two reference data maps were computed according to the data pipeline described above (cf. **Fig. 4(a)**). Each actuator was set to 20% of the maximum stroke while the other actuators stayed in their bias position and response maps were obtained from the recorded pupil plane images. The reference maps were subtracted from the poking responses of the P-WFS and the resulting relative P-WFS poking responses were stored in the interaction matrix. An example of the absolute and relative response of the P-WFS to poking of DM actuator Nr. 25 are shown in **Figs. 4(b)** and **4(c)**.

The AO software for closed-loop correction is configured to enable switching between the input from the P-WFS and the SH-WFS to drive the AO control and the DM actuator command updates are computed by multiplying the slope maps measured for the residual wavefront with the respective control matrix obtained in the calibration. (The calibration procedure for the SH-WFS is described in the **Appendix A.1**). No pre-correction of the system aberrations is applied and the control iteratively adjusts the actuators such that the measured slope maps converge towards zero correcting for all aberrations introduced by the eye and the system. The illuminated pupil is defined via intensity threshold in each AO correction step and might not cover the entire studied pupil considered in the calibration because of eye motion or smaller pupil diameters. For both sensors, the slope values at sample points that lie outside the illuminated pupil are set to zero. A constant loop-gain of 0.25 and an exposure time of 40 ms was applied for both sensors for all *in vivo* measurements. The AO control software has not yet been optimized for speed leading to a correction bandwidth of ∼6 Hz and the AO loop currently converges within a few seconds. To ensure that the entire field of view (FoV) is covered within a single exposure of the WFS, the sampling (number of B-scans) in slow direction is reduced during loop convergence. The measured wavefront aberrations are therefore averaged over the entire FoV and only aberrations that do not change over the FoV are corrected. During recording of the AO-OCT volumes, the sampling in the slow direction is reset to full resolution and the AO correction keeps running. Therefore, the wavefront measurements are now averaged over the full range of the fast scanning direction and only over a part of the slow scanning direction.

An important feature of any AO system for retinal imaging is the capability to set the focus of the imaging beam to distinct layers in the retina as the depth of focus is considerably smaller than the depth extension of the retina. In the used system, only light returning from the retina that is in an orthogonal polarization state (with respect to the incident polarization state) is directed to the wavefront sensors. As the back-scattered light from the RPE is in a random polarization state [43], light originating from this layer will mainly contribute to the wavefront measurement. Thus, the inherent focus of the system at hand is on the outer retinal layers [11]. In order to shift the focus to the anterior layers of the retina, the AO control must drive the sensor data maps not towards zero but towards data maps that represent a defocus wavefront. For the P-WFS, such target sensor data maps were computed via a polynomial fit from sensor data recorded in the model eye for different amounts of defocus which were introduced by displacement of the artificial retina after pre-correction of system aberrations. The P-WFS data maps in **Fig. 4(d)** were measured for a defocus wavefront of 0.4 µm root mean square (RMS) amplitude. A wavefront estimate obtained with the SH-WFS and the corresponding Zernike decomposition of the estimate in **Figs. 4(g)** and **4(h)** confirm that the dominant component in the present aberration was defocus. The linear surface fits to the measured P-WFS data maps, displayed in **Fig. 4(e)**, were used as target for focus shifting with the P-WFS. The polynomial fitting error maps in **Fig. 4(f)** show the components of the measured P-WFS data which were discarded by the linear fit. The low order variations can be attributed to the residual astigmatism modes seen in the Zernike decomposition of the reconstructed wavefront and the variations of higher spatial frequency in the error maps can be explained by the effect of the pupil edges and high order residual aberrations. The presented closed-loop AO routine for inner retina focusing with the P-WFS is a preliminary implementation and in its current form less stable than the routine for outer retina focusing. Therefore, the following procedure is applied: first the wavefront aberrations are corrected using zero maps as target, then the target is switched to the fitted defocus sensor data maps and the loop is stopped once the signal from the anterior layers is sufficiently strong which can be checked via the real time cross sectional view of the retina (OCT-B-scan). Then the data recording is started.

### 2.3 Imaging protocols and subject selection

The system supports an imaging beam diameter of 7 mm and the AO control algorithm automatically adapts to the observed pupil size of the subject. The presented AO-OCT volume data were recorded with scanning angles of 1° x 1° and 4° x 4° for the small and large field of view (FoV) images, respectively. The sampling densities in x and y direction were chosen with the aim of good visualization of the targeted cellular structures while minimizing motion artifacts. A configuration of 300 × 300 pixels (300 A-scans per B-scan, 300 B-scans) was applied for the small FoV imaging, and a sampling of 750 × 750 pixels was used for the images recorded with a large FoV. In z- (depth) direction the number of pixels is constant with 400 pixels according to the used spectrometer configuration (the light spectrum is dispersed over 800 pixels). The processing applied to the recorded spectral data is similar to standard OCT processing and images are shown on a linear intensity grey scale. For all data volumes, axial displacement between B-scans was corrected in post processing. In case of the large FoV, additionally, the curvature of the retina within the B-scans was compensated for and lateral motion between the B-scans was corrected as outlined in detail elsewhere [11]. All but one (the small FoV images of the anterior retinal layers in **Fig. 7**) of the presented sets of images were extracted from single-shot AO-OCT volumes. This specific image set was retrieved from a data volume that was created by registering and averaging of several image volumes in order to reduce speckle noise [12].

Five healthy subjects ranging in age between 22 and 30 years (mean age of 27.2 years) participated in this study (see the first part of **Supplement 1**). The volunteers were recruited with the selection criteria of negligible media opacities and stable fixation and had a spherical equivalent refraction between <−0.25 and −2.00 Diopters. All measurements adhered to the tenets of the Declaration of Helsinki and were performed after approval of the study by the local ethics committee. Written consent was obtained from each subject prior to the measurement after explaining the nature and form of the procedures. A headrest and a manually adjustable XYZ translation stage were used to stabilize and align the eyes and heads of the subjects. No drugs were administered for dilating the pupil since the pupil diameter under the low light conditions in the lab was larger than 6 mm in all subjects. The volunteers were asked to fixate on an internal fixation target which was optically set to infinity for imaging in the fovea and on an external fixation target for imaging in the periphery. Since accommodation of subjects was not prevented, some data sets showed varying focus settings (associated with varying visibility of the targeted retinal layer) and were discarded.

## 3. Results

### 3.1 Imaging of the outer retina in the fovea

**Fig. 5.**
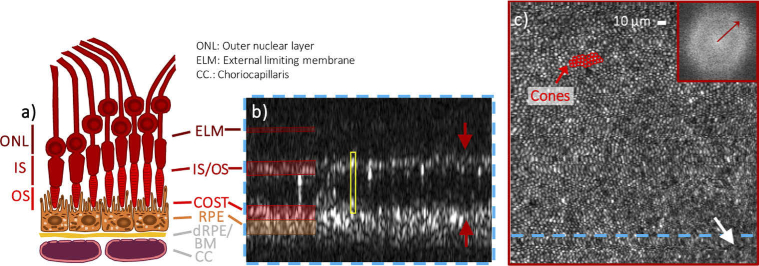
*In vivo* AO-OCT images of cone photoreceptors obtained with AO correction based on the P-WFS. The representative images were retrieved from a single data volume recorded at the fovea of a healthy volunteer (28 years, female, left eye). The field of view is approximately 0.94° x 0.99°. The en-face projection in c) was created by depth integration over the cone photoreceptor layers and is accompanied by the 2D Fourier transform (FFT) which shows a Yellott’s ring. The radius of the Yellott’s ring indicates the spatial frequency of the cone mosaic that corresponds to the row to row spacing of the cones in the imaged areas. The white arrow indicates the approximate location of the fovea centralis (estimated by the highest density of cones). The blue dashed line highlights the location of the single B-scan shown in b), where the yellow box highlights a single cone photoreceptor and the red arrows mark the limits of the en-face integration range. A sketch of the outer retinal bands as known from histology is provided in c).

As a test scenario to demonstrate the repeatability of high performance ophthalmic AO with the P-WFS, we have chosen AO-OCT imaging of the photoreceptor layer at a small FoV of 1° x 1° in the central fovea of 5 healthy subjects with large pupil diameters > 6 (see the first part of **Supplement 1** for subject characteristics). The cell density packing of the cone photoreceptors in the fovea centralis [44], the part of the central fovea where the photoreceptors have the tightest packing, is at the limit of the system’s theoretical transverse resolution of ∼2 µm. For the small FoV imaging, we opted for an OCT sampling of 300 × 300 pixels resulting in a lateral spacing of 1.2 µm. The representative image data displayed in **Fig. 5** show an en-face image of the photoreceptor mosaic of subject V1_R obtained through depth integration over the outer retinal layers containing the cones and a cross-sectional B-scan image extracted at the fovea centralis. The integration range was chosen sufficiently large to include all reflective spots that can be associated with the interface between the inner and outer segments (IS/OS) and with the cone outer segment tips (COST) of single cone photoreceptors. The power spectrum of the en-face image was computed via 2D Fast Fourier transform (FFT) and the radius of the clearly visible Yellott’s rings indicates the spatial frequency that scales with the density of the resolved photoreceptor cells. In the presented data set, single cone photoreceptors can be visualized in the fovea centralis in both the en-face and B-scan image (**Fig. 5(b) + (c)**) demonstrating the excellent AO correction quality obtained with the P-WFS. In the first part of **Supplement 1**, AO-OCT data sets recorded in 5 healthy subjects using the P-WFS are provided. The cone and RPE mosaics could be visualized in the central fovea for all subjects. For subject V1_R and V4_L who have the largest pupil sizes, even the cone photoreceptors in the fovea centralis could be resolved.

We employed the same AO-OCT imaging scenario using the well-known SH-WFS, in order to provide an additional performance benchmark for the AO imaging quality obtained with the P-WFS. Since the cone photoreceptor density increases exponentially towards the fovea centralis, en-face visualizations of the cone mosaic can serve as an excellent resolution target to judge the performance of ophthalmic AO at a constant pupil size. Several data sets were recorded in 8 eyes (both eyes of volunteers V1 to V4) in a single imaging session, respectively, alternating between the P-WFS and the SH-WFS (see **Appendix A.2**). A pupil sampling comparable to the one provided by the SH-WFS was chosen for the P-WFS and comparable standard calibration routines were applied (see **Section 2.2** and **Appendix A.1**). The AO imaging quality achieved with the P-WFS was in all eyes equivalent or even better than the quality obtained with the SH-WFS of the system. In **Appendix A.2**, a quantitative analysis of the *in vivo* AO imaging quality of the sensors is provided, followed by a comparison of the WFSs in terms of sensitivity and an assessment of the P-WFS dynamic range, both performed in a model eye, in **Appendix A.4**.

The AO imaging quality of the P-WFS for larger scanning angles to form a large FoV of 4°× 4° was successfully tested by means of AO-OCT data recorded in the central fovea of several volunteers underlining the capability of the P-WFS to support high resolution imaging at both small and large FoVs. The A-scan density was adjusted to 750 × 750 pixels in order to obtain good visualization of the cone mosaic pattern while minimizing motion artifacts. Representative imaging data obtained with subject V4_L is provided in the second part of **Supplement 1**.

### 3.2 Imaging of the outer retina in the periphery

**Fig. 6.**
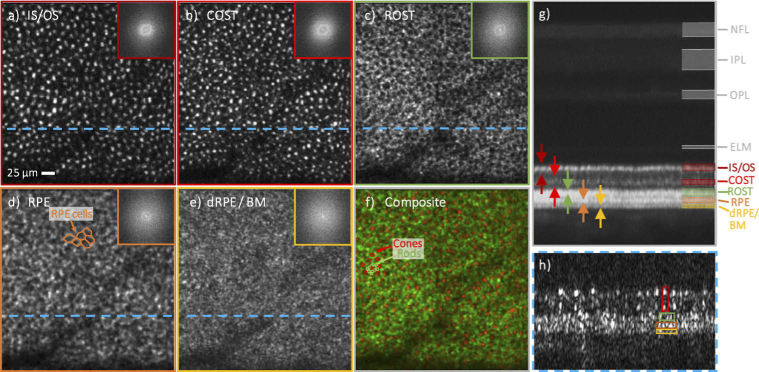
Different cell types in the outer retinal layer recorded at an eccentricity of 14° temporal / 6° superior in a healthy volunteer (29 years, male, right eye) with a FoV of 0.95° x 1° using AO-OCT with the P-WFS. The en-face images, a) to e), were obtained from a single data volume by integration over different depth ranges in the outer retinal band and have the respective 2D FFTs as an inset. The averaged B-scan in g) shows a projection of the entire volume along the slow scanning direction with the arrows indicating the integration ranges for a) to e). For the following en-face projections, specific retinal cell types can be identified as main contributor in terms of reflected signal: a) junction between inner and outer segments of cone photoreceptors (IS/OS), b) cone outer segment tips (COST), c) rod outer segment tips (ROST), d) retinal pigment epithelium cells (RPE). The structure in e) presumptively corresponds to the distal part of the RPE or the Bruch’s membrane (dRPE / BM). The composite in f) is a false color image of COST (red) and ROST (green). In the single B-scan in h), only the outer retinal bands are shown and a selection of the hyper-reflective spots, which form the cell mosaics in a)-d) and the structure visible in e), is marked. The location from which h) was extracted is highlighted in the en-face images with a blue dashed line

In a next step, we recorded AO-OCT data using the P-WFS at an eccentricity of 14° temporal / 6° superior from the fovea of subject V4_R with scanning angles of 1°× 1° and 4°× 4°. **Figure 6** displays the representative AO-OCT imaging data. From a single-shot volume, en-face visualizations at several outer retinal layers, a single B-scan showing only the outer retina and an averaged B-scan including the signal from the inner retina were extracted. The en-face visualizations obtained by integration over the IS/OS (**Fig. 6(a)**) and COST (**Fig. 6(b)**) layers show the coarser packing of the cone photoreceptors at this peripheral imaging location. In the averaged B-scan of **Fig. 6(g)**, three bright bands can be clearly distinguished below the COST layer with the top most corresponding to the rod outer segment tips (ROST). In the en-face visualization of the ROST layer (**Fig. 6(c)**), the rod photoreceptor cells are observed as hyper-reflective spots ordered around hypo-reflective spots marking the locations of the cone photoreceptors. The false color image (**Fig. 6(f)**) is a composite of the en-face images of COST and ROST and visualizes the arrangement of rod photoreceptors that surround cones. The fact that individual rod photoreceptor cells can be resolved, albeit only at a small FoV, in a single-shot AO-OCT volume (see **Figs. 6(c), (f) and (h)**) highlights the excellent performance achieved with the P-WFS. The second highly reflective band below the COST layer stems from retinal pigment epithelium cells (RPE), whose mosaic can be clearly visualized in the en-face image of **Fig. 6(d)**. Note the different spatial frequencies of RPE cells and cone cells as indicated by the varying radii of Yellott’s rings. Posterior to the RPE layer, a third reflective band can be observed which manifests as a granular structure in the en-face visualization and presumptively can be associated with the distal parts [45] of the RPE layer or Bruch’s membrane (**Fig. 6(e)**).

**Fig. 7.**
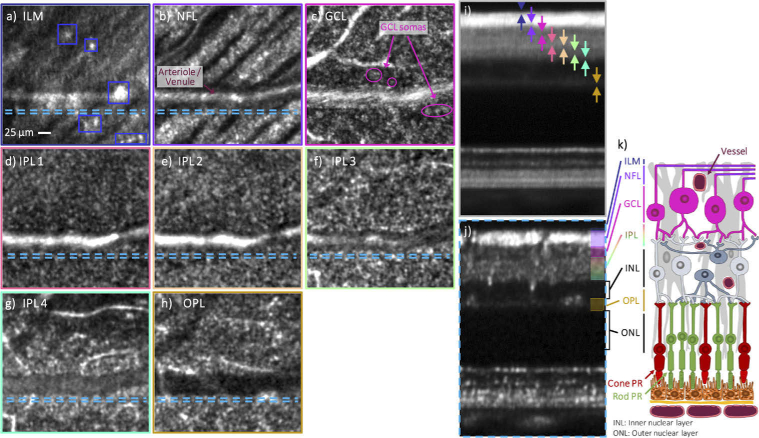
Different structures of the inner the retina visualized for a healthy volunteer (29 years, male, right eye) at 14° temporal / 6° superior with AO-OCT using the P-WFS. The focus was set to the nerve fiber layer and the FoV is 0.82° x 0.78°. The representative images are extracted from a volume obtained by averaging 12 registered volumes that were recorded within ∼40 sec. The en-face images a)-h) were obtained by depth integration over parts of or in vicinity of the following retinal layers: a) inner limiting membrane (ILM), b) nerve fiber layer (NFL), c) ganglion cell layer (GCL), d)-g) inner plexiform layer (IPL), h) outer plexiform layer (OPL). The integration ranges are indicated by color-coded arrows in the averaged B-scan in i) which was obtained by projection of the full data volume in the slow imaging direction. The dashed lines in the en-face images mark the location of the 5 adjacent B-scans averaged in j) in the data volume. k) is a sketch of the retinal layers and cells as known from histology

The AO-OCT image data of the outer retinal bands at the periphery recorded with the large FoV is presented in the second part of **Supplement 1**. With the extended FoV, the different cell mosaics can be visualized over a sufficiently large imaging area to make more conclusive observations about the arrangements of the respective cell types. On the other hand, while the large FoV imaging enables us to see large scale structures like vascularity that are very useful for orientation on the retina, small details might be missed because of the inferior resolution. For example, it can be clearly seen in the small FoV images of **Figs. 6(d) and (h)** that the boundaries of the RPE cells which appear as continuous in the large FoV data are in fact formed by discrete points.

### 3.3 Imaging of the inner retina in the periphery

In a final step we demonstrate the capability of the P-WFS for ophthalmic AO-OCT by imaging inner retinal layers. This requires the application of a specific defocus value to the target wavefront slopes of the P-WFS during AO-correction. The focus of the P-WFS controlled AO was hereby set to the nerve fiber layer (NFL) and AO-OCT data were acquired with small and large scanning angles. For the small FoV imaging of the inner retina, within ∼40 sec, a total of 15 data volumes were recorded in three runs each consisting of resetting the deformable mirror, AO loop convergence and consecutive recording of 5 OCT volumes at a scanning angle of 1° x 1° with a 300 × 300 A-scan sampling. Three volumes were corrupted by microsaccades and were therefore discarded. The remaining volumes were registered using a stripe-wise approach and averaged to improve the signal-to-noise ratio and image contrast in the nearly transparent neuronal layers anterior to the photoreceptor bands. From the resulting averaged data volume, only the part in which all the original volumes overlap was used to create the image data presented in **Fig. 7**. Next to two cross-sectional views, the average of all B-scans (**Fig. 7(i)**) and 5 adjacent B-scans (**Fig. 7(j)**), en-face images are presented visualizing details in the inner retinal bands: On the surface of the inner limiting membrane (ILM), irregular highly reflective structures can be observed (**Fig. 7(a)**) that were associated with macrophage-like cells [46] or microglial cells [47]. In the nerve fiber layer (NFL), single nerve fiber bundles crossed by a large arteriole/venule are visualized (**Fig. 7(b)**). In the ganglion cell layer (GCL), ganglion cell somas of different sizes (8–24 µm) can be distinguished in the lateral view in **Fig. 7(c)** as well as in the cross-section in **Fig. 7(j)**. The relatively small number of AO-OCT volumes (compared to previous work [12]) that were required to visualize ganglion cells attests the high AO imaging performance and sensitivity achieved with the P-WFS based system. Further, en-face images were extracted from four depth locations within and at the outer edge of the inner plexiform layer (IPL) (**Fig. 7(d-g)**), which show dense meshes of high spatial frequency irregularities which have been reported in the literature as dendrites and synapses between GCs, amacrine cells, and bipolar cells [12]. Despite the focus being set to the NFL, the resolution and image contrast in the en-face image in **Fig. 7(h)** is sufficient to visualize details in this transverse slice located in the vicinity of the outer plexiform layer (OPL). Representative imaging data of the inner retina recorded with the P-WFS and an extended FoV of 4°× 4° is shown in the second part of **Supplement 1**.

## 4. Discussion

In this work we demonstrate high performance of a non-modulated pyramid wavefront sensor (P-WFS) to drive an adaptive optics (AO) system in retinal imaging. The configuration was tested *in vivo* in 5 healthy volunteers and stable closed-loop AO correction was achieved reliably. For the first time, ophthalmic AO imaging was performed using a P-WFS that provides state-of-the-art high resolution images of the retina at a cellular level. The obtained excellent imaging quality using AO-OCT for imaging is due to the high quality of the optical system as well as the excellent performance of the P-WFS. It should be mentioned that the subjects’ pupils were not artificially dilated and accommodation was not suppressed leading to the necessity of discarding of some volume data sets and a slight degradation in resolution in subjects with smaller pupil size. The performance of the ophthalmic AO imaging with the P-WFS was demonstrated in various retinal imaging scenarios of interest by displaying en-face and cross-sectional views extracted from AO-OCT volumes. Specifically, the visualization of rods and central foveal cones underlines the performance of AO correction provided by the sensor. The applicability of the sensor was tested by imaging in the central fovea and in the periphery (14° temporal / 6° superior) as well as by shifting the focus of the imaging beam to the inner retinal layers. The sensor is usable for both, small (1°× 1°) and large (4°× 4°) FoV imaging indicating similar variety of applications as is known for the commonly used SH-WFS.

AO correction was achieved in the eye with a standard calibration procedure based on a slope-like P-WFS data definition. Oscillation of the pyramid or modulation of the beam, which is generally applied for the P-WFS to increase the linear regime of the sensor response and subsequently the dynamic range of the sensor was not necessary which greatly simplifies the overall design of the sensor. The non-modulated P-WFS was further implemented with a point source and without the use of a diffusive element. While it is not compulsory to use the P-WFS only in the linear regime if the sensor signals are used to drive closed-loop AO correction, we hypothesize that the excellent and stable AO performance was achieved because the sensor response is to a certain extent linearized by using part of the imaging light for wavefront sensing and the scanning of the imaging beam over a larger area on the retina during P-WFS exposure. As aberrations will be corrected for both, the illumination and detection light paths, the size of the focal intensity distribution (point spread function) at the retina is changed during AO-correction. This intensity distribution, however, serves as source for wavefront sensing. It has been shown that using an extended light source instead of an ideally point-like source for wavefront sensing creates a similar effect as beam oscillation [24, 31]. Larger aberrations, in the eye dominated by defocus and astigmatism, lead to a broadening of the focal intensity distribution at the retina and subsequently at the tip of the pyramid. During convergence of the AO loop, aberrations are corrected for, leading to a sharper focal intensity distribution at the retina and at the sensor, approximating the condition of a point-like source for wavefront sensing. This can be viewed similar to a dynamic beam modulation which is generally adopted to create a large dynamic range with large modulation at the beginning of the correction, and a high sensitivity with small modulation once the loop is close to convergence. The second aspect is that the shape of the focal intensity distribution at the tip of the pyramid is not only determined by wavefront aberrations but by tissue structure (scattering potential of the illuminated area on the retina) as well. During exposure of the P-WFS the beam moves over a larger area of the retina (on its way back to the sensor the beam is de-scanned), thus averaging out the influence of the underlying tissue structure on the shape of the focal intensity distribution at the pyramid. This shape is therefore solely determined by aberrations that are introduced to the light beam on the return path from the retina.

A major asset of the P-WFS is that it provides flexibility in terms of pupil sampling which can be changed optically or by digitally binning the pixels. There are several advantages in the flexible pupil sampling of the P-WFS as compared to the rigid sampling of the SH-WFS for ophthalmic AO imaging. Without changing the main hardware component of the WFS, it is possible to provide optimal pupil sampling for different types of wavefront correctors which for example would be advantageous in woofer- tweeter AO systems [28]. We want to point out that the SH-WFS suffers from centroid errors due to non-uniform lenslet illumination introduced by partly illuminated lenslets at edges of the pupil or intensity variations within the pupil that occur in ophthalmic imaging or when using a single mode optical fiber as light source [21, 48]. Increasing the pupil sampling, which is easily achievable with the P-WFS and would require new, more expensive hardware for the SH-WFS, is one option to mitigate for the error introduced by intensity variations across lenslets [21]. During *in vivo* imaging, the subjects show differing pupil sizes and shapes which are often smaller than the system pupil size. The approach of filling missing SH-WFS and P-WFS data points with zero, which was also adopted in our WFS implementations, is suboptimal but frequently implemented [20]. A higher pupil sampling available with the P-WFS would naturally reduce artifacts introduced by this approach [21]. Next to irregular pupils, eye movements pose another challenge to AO imaging, especially in clinical applications. Pupil tracking based on SH wavefront sensing data has been proposed previously [49] and could be performed with higher precision using the densely sampled pupil images provided by the P-WFS before binning and without affecting the AO-correction performance. Finally, by implementing a zoom optical relay it is possible to change the P-WFS pupil sampling in a continuous manner [13]. This would allow for a WFS based study of various spatial frequencies present in ocular wavefront aberrations and might give insight into the optimal wavefront corrector required for the application. While it is possible to model the low order aberrations present in the eye, simulation of higher order aberrations as they are, e.g., introduced by the tear film have been harder to predict.

In our initial comparison of the implemented P-WFS and SH-WFS, the presented images of central foveal cones recorded in 5 subjects with both sensors show reliably better results achieved with the P-WFS, when using comparable pupil samplings and standard alignment and calibration procedures. At the current stage, this superiority cannot be generalized and requires further experiments for confirmation since further optimization might be possible for both sensors. Nevertheless, these results give a positive outlook on future studies and motivate the consideration of the P-WFS as standard wavefront sensing device for AO in ophthalmic imaging.

In terms of temporal dynamics, studies have shown that correction bandwidths of ∼30 Hz are required to sufficiently correct lower order ocular wavefront aberrations [50, 51]. Higher bandwidths are essential to follow fast temporal dynamics of higher order aberrations but this requires that more light is dedicated for wavefront sensing which comes at the cost of lower imaging sensitivity. Our initial results obtained in a model eye confirmed studies from astronomical AO [22, 24] which suggest a better performance of the P-WFS in comparison with the SH-WFS in the case of a low light scenario which might be beneficial for improving the AO bandwidth while maintaining high imaging sensitivity. An in-depth comparison of the sensors for the context of ophthalmic imaging needs to be subject of future studies. Depending on the outcome of these studies, the P-WFS might on the long term replace the SH-WFS in visual science similar to what can be observed in astronomy.

An initial assessment of the P-WFS dynamic range in a closed-loop AO correction obtained in a model eye suggest that it will be possible to perform P-WFS based AO imaging in a large part of the population. For subjects with very high spectacle prescriptions, AO correction after pre-correction with prescriptions lenses or glasses is a solution as has been demonstrated.

For the presented P-WFS, a standard, low-cost glass pyramid was used instead of a high precision component as required in astronomy. Since for the P-WFS the detection of the wavefront sensing light is not at a focused but a diffused plane, the requirements on the dynamic range of the cameras can be eased, further adding to the low-cost character of the suggested WFS. Finally, we want to point out that the P-WFS concept presented in this paper can be directly translated to confocal scanning laser microscopy for sensor based AO correction.

## Figures and Tables

**Fig. 8.**
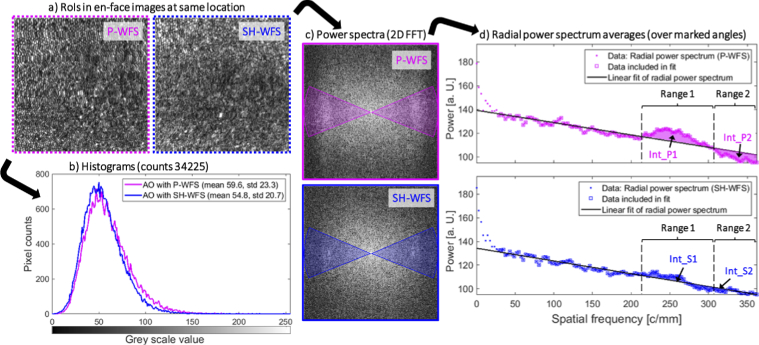
Quantitative comparison of the P-WFS and the SH-WFS by retrieving AO imaging performance metrics from en-face AO-OCT images of the cone photoreceptor mosaic recorded in the central fovea of healthy volunteers: a) shows the common regions of interest (RoIs) defined as close as possible to the fovea centralis. For the RoIs, histograms in b) and power spectra (2D FFT) in c) are obtained. The power spectra are radially averaged over the angles marked in c) and fitted with a linear polynomial in d). The final performance metrics are the standard deviation of the RoIs provided by the histograms and the values obtained by integrating the difference between the radial power spectrum averages and the respective fits over the spatial frequency ranges Range 1 and Range 2.

**Fig. 9.**
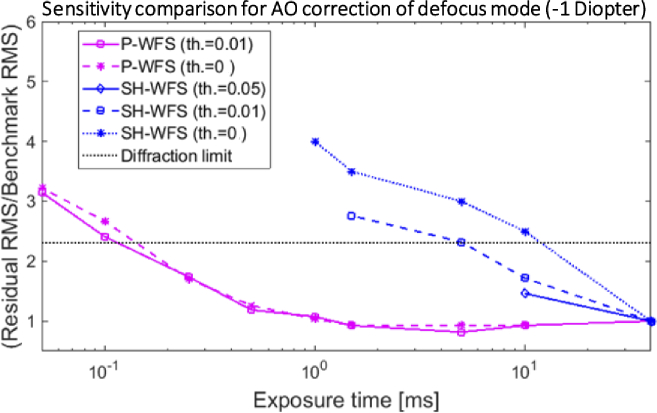
Comparison of the P-WFS and SH-WFS sensitivity. The ratio between the residual wavefront RMS error after AO loop convergence (measured with the SH-WFS at 40 ms exposure time) and the benchmark RMS value is plotted for different exposure times. Several intensity thresholds are considered for the center of gravity computation of the SH-WFS focal spots and the P-WFS pupil image masking. The benchmark RMS value corresponds to the residual wavefront RMS value after AO loop convergence obtained for the respective sensor setting at 40 ms exposure time. The dashed horizontal line marks the performance required for diffraction limited imaging according to the Maréchal criterion.

## Data Availability

Data underlying the results presented in this paper are not publicly available at this time but may be obtained from the authors upon reasonable request.

## Appendix A

### A.1 Components and calibration of the SH-WFS

The customized SH-WFS was described in detail earlier [35]. It consists of a lenslet array with 32 × 32 micro-lenses (Adaptive Optics Associates C-0300-16-S) that is located in a pupil plane of the system. The focal spots formed by the lenslets are recorded with a CMOS camera (Pixelink PL-A741). The focal length of the lenslet array is 16 mm and the lenslet pitch is 300 µm. A commercial adaptive optics software, Alpao Core Engine, running in MATLAB is used to compute the centroids of the SH-WFS focal spots which provide a sampling of 22 across the pupil diameter. The specifications of the P-WFS and the SH-WFS used in this work are summarized in **Table 1**.

**Table 1.** Specifications of the pyramid and the Shack-Hartmann wavefront sensors

	P-WFS	SH-WFS
Pixel size of the detector	5.5 µm	6.7 µm
Dimension of the detector	2040 × 1088 pixels	1180 × 1024 pixels
Bit depth of the detector	8	10
Sampling of the pupil plane (considered in the experiment)	flexible (digitally binned pixels)	22 × 22 subapertures

For calibration of the SH-WFS, we adapted the procedure from [35] to match the calibration steps for the P-WFS presented in **Section 2.2**. Instead of using the original calibration of the SH-WFS for closed-loop correction, that had been performed with a flat mirror at the eye pupil location and without scanner motion, the calibration was performed in the model eye, with moving scanners and with the same pre-correction applied (to minimize system aberrations). The same zonal control approach as for the P-WFS using the same number of controllable modes was applied for AO correction with the SH-WFS. As for the P-WFS control, an intensity threshold was applied and the outer ring of partially illuminated subapertures was excluded from the mask which defines the studied pupil (this step was also included in the original SH-WFS calibration). Since after the pre-correction of the system aberrations, the SH-WFS sees a plane wavefront there is no need for the acquisition of a new reference frame. Note here a difference to the P-WFS, which in addition sees the non-common path aberrations between the two sensors (even if those are small), and therefore requires a respective reference frame. For construction of the SH-WFS interaction matrix, a centroid map was acquired for each actuator poke and translated to two wavefront gradient maps using the Alpao Core Engine. The SH-WFS control matrix is also obtained via computation of the pseudo-inverse of the resulting interaction matrix.

### A.2 In vivo benchmarking of the P-WFS using a SH-WFS

For the *in vivo* comparison of the AO imaging quality provided by our non-modulated P-WFS and the SH-WFS, AO-OCT images of the cone photoreceptor mosaic at the central fovea were recorded at a small FoV of ∼ 1° x 1° using both sensors in a single imaging session. The experiment was repeated in 8 eyes (both eyes of volunteers V1 to V4). By adjusting the digital binning, the number of samples used by the P-WFS was set to a pupil sampling of 21 × 21 which is comparable to the 22 × 22 sampling used by the SH-WFS.

In order to avoid any possible bias in favor of the P-WFS regarding tear film degradation or decrease of the pupil size due to an extended stay in a dark environment (i.e. loss in resolution), we first recorded the data with the SH-WFS. To account for motion artifacts or small misalignments of the subject, and thus minimize their influence on the comparison, the following imaging protocol was used: For each sensor, the loop was opened and closed 3 times. After every loop convergence, 5 AO-OCT volumes were recorded in a row, resulting in a total of 15 AO-OCT volumes per sensor. The AO loop kept running during acquisition. One entire imaging session covering both sensors took ∼ 2 min. Volumes affected by motion, micro-saccades or accommodation were discarded, resulting for all considered eyes in at least 5 remaining volumes for each sensor that were of identical aberration correction quality, respectively. From the remaining valid data sets, en-face images showing the cone mosaic were created and identical regions of interest (RoIs) of 0.6° x 0.6°, located in or as close as possible to the fovea centralis, were chosen in both en-face images (see **Fig. 8(a)**). For these RoIs the power spectra were computed via 2D-FFT and compared. For each considered eye, the imaging quality in all valid volumes obtained with the P-WFS was better than the imaging quality of all volumes obtained with the SH-WFS. We observed a clearer appearance of the photoreceptor cells which manifests itself in the higher clarity of the Yellott’s ring obtained for images recorded with AO using the P-WFS.

In a next step, we performed a quantitative analysis of the AO imaging data obtained with the P-WFS and the SH-WFS in terms of imaging contrast and spatial frequency information via observing the radial average of the Yellott’s rings in the corresponding power spectra. In **Fig. 8**, the procedure to obtain the chosen performance metrics from the RoIs (**Fig. 8(a)**) and the corresponding power spectra (**Fig. 8(c)**) is visualized for volunteer V1_R. For the depiction in **Fig. 8**, the contrast was adjusted in the RoI images and the power spectra. The quantitative analysis was performed with the unaltered data. Histograms of the grey scale values (0–256) were computed for the RoIs (**Fig. 8(b)**) which show higher standard deviation (and thus contrast) for the en-face image obtained with the P-WFS than for the en-face image obtained with the SH-WFS. In the power spectra (**Fig. 8(c)**), the Yellott’s rings are washed out along the slow scanning axis due to motion. To account for this, the computation of the radial averages of the power spectra was performed only for the angles marked by the cones in **Fig. 8(c)**. It can be clearly seen in **Fig. 8(d)**, that, for the P-WFS data, power is shifted from the high spatial frequency range (> 310 c/mm) to the spatial frequency range which corresponds to the expected range of cone photoreceptor row-to-row spacing. This power shift is significantly less pronounced for the SH-WFS data, suggesting that the smaller cone photoreceptors are not resolved and instead washed out into noise of high spatial frequency. In order to quantify this power shift, linear polynomial fits were performed for the two radially averaged power spectra. For the different subjects, the RoIs were always located in the central fovea but at slightly different eccentricities. The improved imaging resolution obtained with the P-WFS resulted in clear Yellott’s rings for all subjects. The spatial frequencies present in the imaged cone mosaic sections could be identified by the cross-over points of the radial power spectrum averages and the corresponding linear fits (see dashed line in top plot of **Fig. 8(d)**). These cross-over points create two spatial frequency ranges for each subject: Range 1, corresponding to the spatial frequencies observed in the cone mosaic resolved with the P-WFS, and Range 2, corresponding to higher spatial frequencies. As a metric for the observed spatial frequencies, integration of the differences between the averaged radial power spectra and the corresponding linear fits was performed over Range 1 and Range 2 for the P-WFS and the SH-WFS data, resulting in the values (Int_P1, Int_P2) and (Int_S1, Int_S2), respectively. Values of Int_P1 and Int_S1 far above zero and values of Int_P2 and Int_S2 far below zero indicate a clear Yellott’s ring and therefore excellent AO imaging quality for the respective data set.

In **Table 2**, the quantitative comparison of the P-WFS and SH-WFS AO imaging quality is summarized for all considered subjects with the superior value for each metric printed in bold and the eye in which the data was recorded indicated by _L and _R. The table shows that the P-WFS outperforms the SH-WFS for 6 out of 8 eyes in terms of image contrast (std of the grey values of the image). For the measure of resolved spatial frequency information, the P-WFS showed highly superior values in 7 out of 8 eyes. The low values obtained for subject V3_L indicate an overall poorer visibility of Yellott’s ring and the difference between the sensors lies within the error margin. Overall, the quantitative assessment of the AO imaging quality confirms a superiority of the P-WFS to the SH-WFS as implemented in our system.

**Table 2.** Quantitative comparison of the P-WFS and the SH-WFS (all metrics given in a. U.)

Volunteer	V1_L	V1_R	V2_L	V2_R	V3_L	V3_R	V4_L	V4_R

Hist. (P): (mean/std)	(564.2 / **27.4**)	(59.6 / **23.3**)	(73.8 / **31.1**)	(69.6 / **32.5**)	(71.4 / **26.8**)	(60.7 / 22.7)	(59.0 / **24.2**)	(58.2 / 24.4)
Hist. (SH): (mean/std)	(55.9 / 22.4)	(45.8 / 20.7)	(78.4 / 28.0)	(88.9 / 31.2)	(53.6 / 20.8)	(81.7 / **30.7**)	(63.8 / 23.8)	(83.9 / **30.8**)

P. Sp. (P): (Int_P1/ Int_P2)	**(544 / −319)**	**(497 / −287)**	**(894 / −489)**	**(829 / −454)**	(46 / −28)	**(406 / −224)**	**(373 / −207)**	**(497 / −287)**
P.SP. (SH): (Int_S1/ Int_S2)	(346 / −198)	(108 / −37)	(1 / −30)	(160 / −86)	**(80 / −39)**	(66 / −38)	(175 / −122)	(−4 / 0)

### A.3 Initial study of the P-WFS sampling flexibility in vivo

To highlight the sampling flexibility of the P-WFS we performed AO imaging (in V1_R and V2_R) when using different pupil samplings and compared the results with data obtained with the SH-WFS. The different pupil samplings were realized digitally by changing the amount of digital binning. The following 4 pupil samplings were tested in a single imaging session: 391 × 391 (no digital binning); 37 × 37, 21 × 21, 13 × 13. The AO loop converged in a stable manner and comparable P-WFS based AO imaging quality was reliably achieved with all samplings. We can therefore conclude that in the presented configuration, the P-WFS shows high performance independent of the pupil sampling. Considering AO correction bandwidth, we however do not recommend to use the settings where no binning is applied. From application in astronomical AO, it is known that oversampling of the P-WFS allows to mitigate possible performance loss due to pupil position misalignments [52]. We therefore used the 37 × 37 pupil sampling option for the recording of the AO-OCT data provided in the main manuscript and the supplementary material. The 21 × 21 pupil sampling is comparable to the pupil sampling provided by the SH-WFS and was used for the comparison study presented above. The sampling of 13 × 13 is the lowest sampling with which a sufficient number of sampling points is obtained to achieve high resolution images with a 69 actuator DM [5]. While these initial results provide some insight on the flexibility of the P-WFS in terms of pupil sampling, a more thorough study is required to draw final conclusions on the optimal pupil sampling for ophthalmic imaging.

### A.4 Initial study of the P-WFS sensitivity and dynamic range in a model eye

Next to the quantitative assessment of the *in vivo* AO imaging quality, we further performed a comparison of the P-WFS and SH-WFS in terms of sensitivity and an assessment of the P-WFS dynamic range in a model eye. The model eye consists of a lens with 30 mm focal length and a scattering surface which can be axially moved, serving as artificial retina. It was verified that the light power incident at both sensors is equal and the pupil sampling of the P-WFS matched the pupil sampling of the SH-WFS. Since the artificial retina shows stronger scattering than the human retina, the light power was reduced at the source to obtain similar power in the WFS arm as in an *in vivo* imaging scenario. Defocus aberrations were introduced by shifting the artificial retina out of the lens’ focal plane that are then corrected by the deformable mirror driven either by the P-WFS or the SH-WFS.

The sensitivity provided by each WFS was assessed by correcting for a constant level of defocus (1 Diopter) at different exposure times ranging from 0.025 ms to 40 ms. The residual wavefront RMS value after loop convergence was then measured using the SH-WFS with a 40 ms exposure time. In **Fig. 9** the ratio between the obtained residual RMS values and a benchmark, which consists of the residual RMS value obtained for the respective sensor setting at 40 ms exposure time, is plotted for the different exposure times. An AO correction is considered as failed if the residual RMS value is larger than 2 times the diffraction limited RMS value (computed with the Maréchal criterion). Different levels of intensity thresholding were considered for the center of gravity (CoG) computation of the SH-WFS focal spots and for the masking of the P-WFS pupil images before applying digital binning. We observed that changes in the CoG threshold highly impacted the SH-WFS behavior for reduced exposure times, while the intensity thresholding on the P-WFS data did only marginally affect the AO correction performance achieved for each exposure time level. For intensity thresholds larger than zero, the SH-WFS provided diffraction limited AO correction down to 5 ms exposure time. The P-WFS was more robust to low flux scenarios and showed no significant loss in AO correction performance down to 0.5 ms exposure for both threshold settings. Loop instabilities due to discarded WFS samples and a slowdown in loop convergence were observed for exposure times < 10 ms and < 0.25 ms for the SH-WFS and the P-WFS, respectively. Applying a zero threshold resulted in a highly unstable AO loop for the SH-WFS, while it had no such effect on the P-WFS performance. A zero threshold is not recommendable for an *in vivo* application of the P-WFS because changing pupil sizes have to be taken into account. We conclude that in our system the gain in limiting exposure time of the P-WFS is at least of one order of magnitude if compared to the SH-WFS. It should be noted that different CMOS detectors are used, however the detector area dedicated to the computation of each pupil sample is of the same order of magnitude for both sensors. An explanation for the different behaviors of the sensors for small exposure times possibly lies in the underlying principle of the sensors which are of very distinctive nature. A more detailed analysis of the matter is beyond the scope of this work and should take into account other low order aberrations and high order aberration, as well as the impact of the CMOS camera specifications on these results.

We further added an assessment of the P-WFS dynamic range by stepwise increasing the defocus aberrations and observing the residual wavefront after convergence of the AO correction. The exposure time was set to 40 ms and the intensity threshold to 0.01. Myopic and hyperopic eyes were modelled by introducing negative or positive defocus aberrations through shifting the artificial retina. Within the range of −3.5 Diopter to 3.5 Diopter, the P-WFS successfully corrected the defocus aberrations without showing a significant decrease in AO correction performance for increasing aberration strength. For aberrations of ±2 Diopter or stronger, the loop gain had to be adjusted in order to obtain convergence and the convergence time increased. If a similar dynamic range can be confirmed for other higher order aberrations in future work, it would be possible to perform P-WFS based AO imaging in a large part of the population. For persons with very large low order aberrations, AO correction after pre-correction with prescriptions lenses or glasses is a solution as has been demonstrated for volunteer V5* in the first part of **Supplement 1**.

## Supplemental document

See Supplement 1 for supporting content.
