# Supplementary material for: Retinal adaptive optics imaging with a pyramid wavefront sensor
Source: Biomed Opt Express. 2021 Sep 2;12(10):5969–90. doi: 10.1364/BOE.438915 (PMC8548025; doi:10.1364/BOE.438915)
Supplement: Supplementary file 1 [file boe-12-10-5969-s001.pdf]

Supplemental Document

Biomedical Optics EXPRESS

Retinal adaptive optics imaging with a pyramid wavefront sensor: supplement

ELISABETH BRUNNER,[1,*] JULIA SHATOKHINA,[2] MUHAMMAD FAIZAN SHIRAZI,[1] WOLFGANG DREXLER,[1] RAINER LEITGEB,[1] ANDREAS POLLREISZ,[3] CHRISTOPH K. HITZENBERGER,[1] RONNY RAMLAU,[2,4] AND MICHAEL PIRCHER[1]

[1] *Center for Medical Physics and Biomedical Engineering, Medical University of Vienna, Waehringer Guertel 18-20, A-1090 Vienna, Austria*
[2] *Johann Radon Institute for Computational and Applied Mathematics, Altenbergerstrasse 69, A-4040 Linz, Austria*
[3] *Department of Ophthalmology and Optometry, Medical University of Vienna, Waehringer Guertel 18-20, A-1090 Vienna, Austria*
[4] *Johannes Kepler University Linz, Industrial Mathematics Institute, Altenbergerstrasse 69, A-4040 Linz, Austria*
[*] *a.elisabeth.brunner@meduniwien.ac.at*

This supplement published with The Optical Society on 2 September 2021 by The Authors under the terms of the Creative Commons Attribution 4.0 License in the format provided by the authors and unedited. Further distribution of this work must maintain attribution to the author(s) and the published article's title, journal citation, and DOI.

Supplement DOI: https://doi.org/10.6084/m9.figshare.15172746

Parent Article DOI: https://doi.org/10.1364/BOE.438915

Retinal adaptive optics imaging with a pyramid wavefront sensor: supplemental document

1. P-WFS based AO for imaging healthy volunteers

Table S1 gives a summary of the volunteers' characteristics. The volunteers were chosen with a natural pupil diameter between 6 mm and 7 mm in dark environment and no drugs for artificial pupil dilation were administered. The pupil diameters along the x and y axis of the pupil plane were estimated from the P-WFS pupil images before digital binning and reveal not only variations in size but also in the shape of the pupils. The RMS amplitudes of the wavefront aberrations were measured with the SH-WFS. The wavefront reconstruction was performed with the Alpao Core Engine using a modal approach [1]: Accommodation was not prevented during the measurements which lead to a slight variation in the measured RMS amplitudes of the wavefront estimates. Therefore, the means of the measured wavefront amplitudes are provided. The standard deviation in the values stayed < 0.7 µm for all considered eyes. The RMS wavefront amplitudes measured for the volunteers extended over a considerable range from ~0.3 µm to almost 1 µm. All subjects showed significant to strong high order aberrations. Volunteer V5 was wearing a contact lens for both the wavefront aberration measurement and the volume recording. Additionally, a routine clinical eye examination including eye length measurements with the ZEISS IOLMaster 500 (Carl Zeiss Meditec AG, Jena, Germany) was performed for each volunteer before the AO-OCT measurement. The mismatch for the Diopter (measured with a Refrakto Keratometer ARK1-S, NIDEK, Gamagōri, Japan) and wavefront aberration RMS values in some subjects is due to the non-linearity of open-loop wavefront measurements with the SH-WFS for larger Diopter values. The imaging data presented in the main manuscript were recorded with volunteers V1 and V4.

Table S1. Characteristics of healthy volunteers included in the study

Volunteer	V1	V2	V3	V4	V5*
Age [years]	28	30	27	29	22
Gender	Female	Male	Female	Male	Male
LE: Pupil diameter [mm]	6.69 x 6.74	6.14 x 6.16	6.05 x 6.44	6.91 x 6.83	
LE: Diopters (Sphe Asm)	-0.25+0.25	-2.00+2.50	-1.75+0.75	<0.25+0.50	/
LE: Wavefront aberration [µm RMS]	0.496	0.801	0.941	0.439	
RE: Pupil diameter [mm]	6.91 x 6.91	6.48 x 6.13	6.05 x 6.30	6.74 x 6.65	6.14 x 6.21
RE: Diopters (Sphe Asm)	-0.50+0.25	-1.50+2.00	-1.25+0.25	<0.25+0.00	<0.25+0.00
RE: Wavefront aberration [µm RMS]	0.752	0.792	0.936	0.556	0.350
Explanations: LE refers to the left eye and RE to right eye of the subject. In the main text, the eye is indicated by _L and _R, respectively. If marked with *, the subject was wearing a contact lens.					

In **Fig. S1**, we provide representative AO-OCT image data recorded in the fovea of 5 healthy subjects to demonstrate the applicability of the P-WFS for AO imaging in more subjects. The data was acquired at a small field of view FoV (scanning angles of 1° x 1°) and a OCT sampling of 300 x 300 A-scans. All images were obtained from single-shot volumes, no volume averaging was applied. Note that Yellott's rings (insets in **Fig. S1**) are more blurred along the slow imaging direction because eye motion mainly distorts structures in this direction.

For volunteers V1 and V4, the photoreceptors could even be resolved in the most central part of the fovea centralis where the cones are most densely packed (cf. **Figs. S1a1** and **S1a4**). The larger pupil diameter sizes of V1 and V4 yield a better theoretical lateral resolution but

also increase the impact of wavefront aberrations on the retinal images. The visualization of the most central foveal cone photoreceptors is therefore a telltale sign of an excellent AO correction quality obtained with the P-WFS. In all presented cross-sectional views, the cone photoreceptor layers do not appear as continuous bands but as rows of discrete points corresponding to the junctions of IS/OS and COST of single cone photoreceptors. In the layer of the cone OS, more sparsely distributed highly reflective spots can be observed.

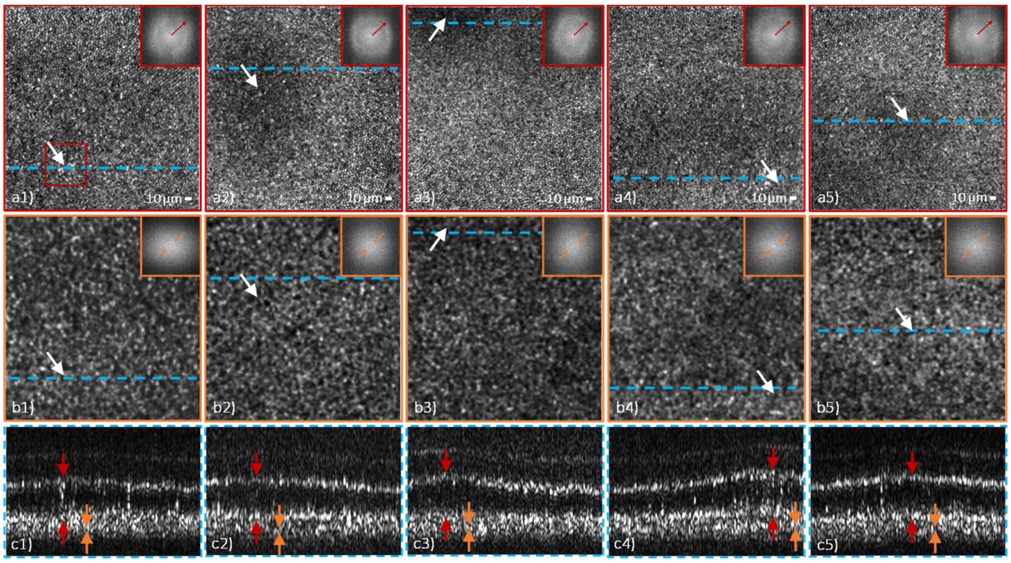

Fig. S1. Representative AO-OCT images recorded in the central fovea of 5 healthy volunteers with the P-WFS and a field of view of 0.94° x 0.99°. The numbers in the image labels indicate the subject numbers that are also shown in Table S1 (data sets 1-3 and 5 were recorded in the right eye and data set 4 in the left eye of the respective subject). The en-face images in a1)-a5) and b1)-b5) were retrieved from single data volumes by depth integration over the cone photoreceptor layers and the layer of retinal pigment epithelial cells (RPE), respectively. The radii of Yellot's rings in the corresponding 2D Fourier transforms (FFT) indicate the spatial frequencies of the cone and RPE mosaics that correspond to the row to row spacing of the cones and RPE cells in the imaged areas. The blue dashed lines highlight the locations of the single B-scans shown in c1) -c5), which were extracted in the fovea centralis The white arrows point to the approximate location of the fovea centralis (estimated by the highest density of cones). The red and orange arrows mark the limits of the en-face integration ranges.

In the en-face images of the RPE (**Figs. S1b1 – S1b5**), the characteristic mosaic of RPE cells can be clearly identified. The hexagonal shapes of the RPE cells are formed by hyper-reflective discrete points which most likely originate from scattering organelles in the RPE cells [2]. Direct imaging of RPE cells remains a challenge because of their low intrinsic contrast and typically extensive averaging of several volumes is used to enhance cell contrast taking advantage of cell motility [2]. We previously demonstrated single volume AO-OCT imaging of RPE cells at a large FoV of 4° x 4°with the SH-WFS [3]. The extended FoV improves the visibility of the Yellott's ring since more RPE cells are visualized and contribute to the generation of the ring. In this work, faint Yellott's ring could be visualized for single volume AO-OCT images of the foveal RPE layer recorded at a small FoV of 1° x 1° highlighting the exceptional AO performance achieved by the P-WFS. The Yellott's rings in the power spectra of the RPE mosaics have smaller radii than for the cone photoreceptor mosaics due to the larger row to row spacing of the RPE cells. Further, their appearance is sharper since the row to row spacing of the RPE cells is constant throughout the field of view. The spacing of the foveal RPE cells was quantified for all volunteers by computing the radial average of the respective power spectra. The determined row to row spacings (14.1 µm, 13.7 µm, 12.7 µm, 12.4 µm and 12.6 µm for subjects V1, V2, V3, V4 and V5, respectively) are in the expected range [2, 3].

The density of the cone photoreceptor packing increases exponentially towards the fovea centralis and a range of spatial frequencies are observed resulting in a broader Yellott's ring for the photoreceptor layers. For subject V1, the row to row spacing of the cone photoreceptors in the fovea centralis was obtained in the same manner from the power spectrum computed for the area (0.2° x 0.2°) marked in the en-face image of **Fig. S1a1**). The resulting row to row spacing of 2.65 µm, which (assuming a hexagonal cone packing) corresponds to a next neighbor spacing of 3.06 µm, is in good agreement with literature [4, 5].

2. Imaging of the retina in the periphery with an extended field of view

In **Figs. S2** to **S4**, representative AO-OCT image data recorded at a large FoV with scanning angles of 4° x 4° are shown. The data presented in **Fig. S2** was recorded in the central fovea of volunteer V4_L and the data presented in **Figs. S3** and **S4** at the periphery of V4_R. The focus of the P-WFS based AO was set to the outer retina (more specifically, the RPE layer) for the first and second volume and to the inner retina (more specifically, the NFL layer). All three data sets are single AO-OCT volumes acquired with a 750 x 750 A-scan sampling.

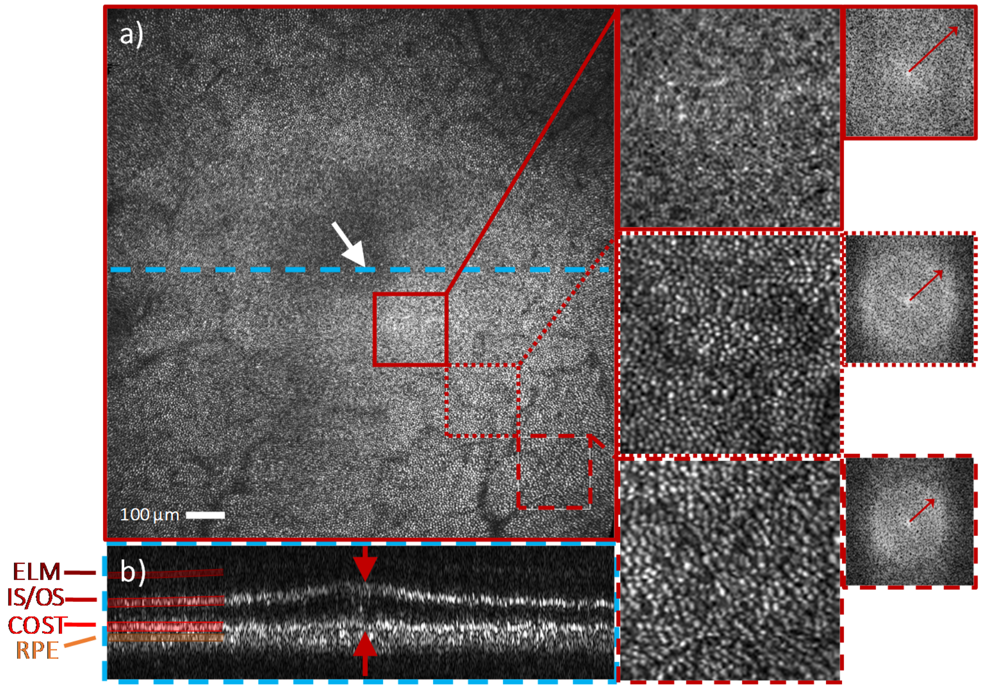

Fig. S2. Representative AO-OCT images recorded in the fovea of a healthy volunteer (29 years, male, left eye) with the P-WFS at an extended field of view of 3.79° x 3.92° (corresponding to 1.36 mm x 1.4 mm on the retina). The en-face image in a) was retrieved from a single data volume by depth integration over the cone photoreceptor layer with the integration range indicated by red arrows in the B-scan image in b). The B-scan location is marked by the blue dashed line in a) where the white arrow points to the approximate location of the fovea centralis. The three en-face images on the right are magnified views of the boxed areas in a). The radii of Yellot's rings in the adjoining 2D FFTs indicate the respective spatial frequencies of the cones at different eccentricities from the fovea centralis.

In the en-face visualization extracted from the data of the outer retina in the central fovea (**Fig. S2a**), individual photoreceptors can be resolved as close as to a distance of ~0.2° from the fovea centralis. Unlike for the small FoV (see **Fig. 5** in the main manuscript), the photoreceptor cells could not be visualized directly in the fovea centralis when imaging with the large FoV. This can be explained by two factors that are independent of the WFS type: Firstly, the lateral spacing between A-scans is relatively large (~1.9 µm) which leads to a slight under-sampling

of the small cone photoreceptors in the fovea centralis. Secondly, despite the optimized system configuration, a compromise is made in AO correction quality when imaging an extended FoV because the wavefront measurements are now averaged over a larger area. The en-face image in **Fig. S2a**) confirms the large field capabilities of the P-WFS. In the enlarged regions of interest (RoIs) and the corresponding 2D FFTs, it can be observed how the packing density of the cell mosaic increases with decreasing distance from the fovea centralis which reflects in the larger radii of the Yellott's rings.

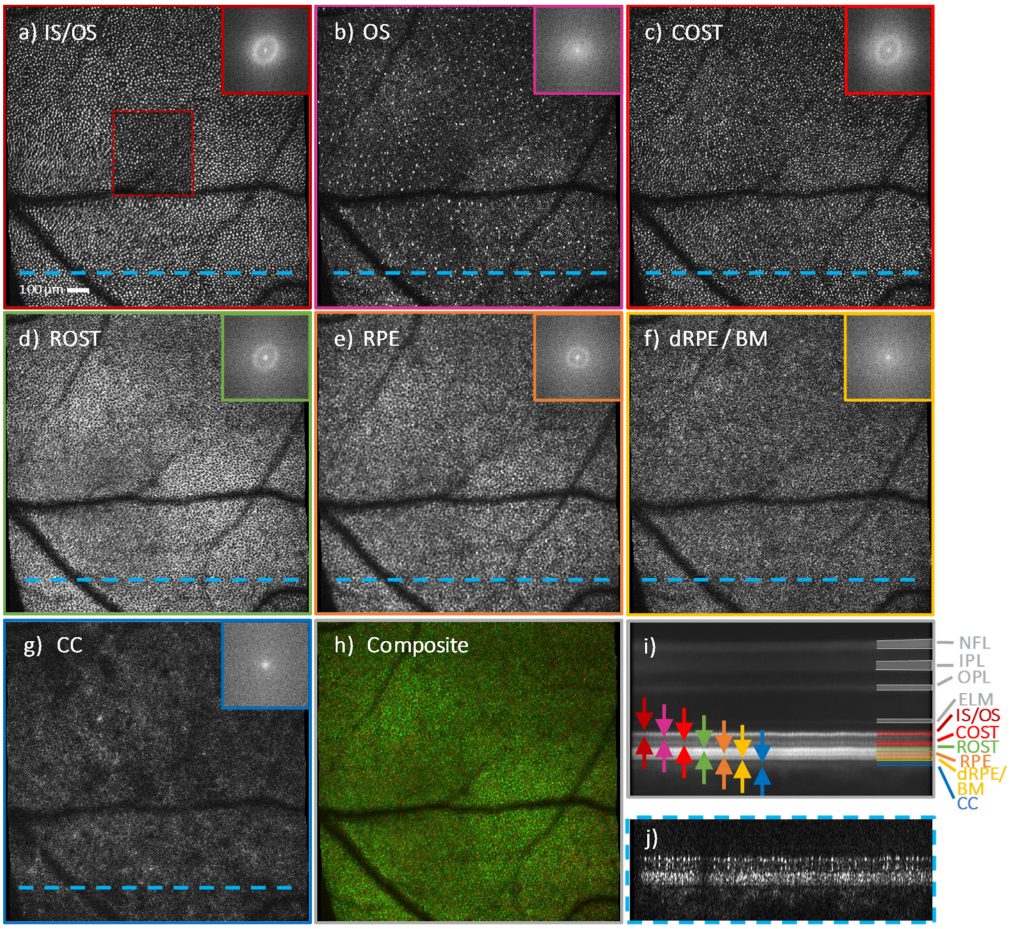

Fig. S3. Visualization of outer retinal layers using AO-OCT with the P-WFS at an extended FoV of 3.99° x 3.92°. The imaging location is 14° temporal / 6° superior in a healthy volunteer (29 years, male, right eye). The representative en-face images a)-h) are obtained from a single data volume by integration over layers in the outer retina and accompanied by their 2D FFTs in insets: a) junction between inner and outer segments of cone photoreceptors (IS/OS) with the small FoV image of Fig. 5. a) as inset, b) hyper-reflective spots in the cone outer segment layer (OS), c) cone outer segment tips (COST), d) rod outer segment tips (ROST), e) retinal pigment epithelium cells (RPE), f) presumptively the distal part of the RPE or Bruch's membrane (dRPE / BM), g) choriocapillaris (CC), h) composite false color image of COST (red) and ROST (green). The integration ranges are indicated by the color-coded arrows in the averaged B-scan i) which consists of a projection of the entire volume along the slow imaging axis. The location of the single B-scan in j) in the same data volume is marked by the dashed lines in the en-face images.

The large FoV AO-OCT imaging data of the outer retina is presented in **Fig. S3**. In the en-face visualizations, the depth span from IS/OS to COST was divided into three integration ranges. The highly reflective spots appearing in the additional en-face image created over the

outer segments (OS) of the cone photoreceptors (cf. **Fig. S3b**) have been reported before [6] and probably correspond to defects in the packing arrangement of the cone outer segment discs. Compared to the small FoV data (see **Fig. 6** in the main manuscript), the clarity of Yellott's rings observed in the power spectra of the presented en-face images increases for the larger FoV as more cells are included in the analysis. At this eccentricity the IS/OS, COST and ROST layer show an elliptic Yellott's ring (cf. insets of **Figs. S3a**, **S3c** and **S3d**), which indicates that the packing density along a radial line from the fovea of the cone photoreceptors is higher than along the orthogonal direction. In contrast to this observation, the Yellott's ring of the RPE mosaic is perfectly circular (cf. inset of **Fig. S3e**), indicating a more homogenous packing arrangement of these cells. Structures of high sparsity or low signal strength, as for example the reflective spots on the OS layer or the vessel mesh of the choriocapillaris (CC), that might not be recognized as such with a small FoV are revealed in the large FoV images (cf. **Figs. S3b** and **S3g**, respectively).

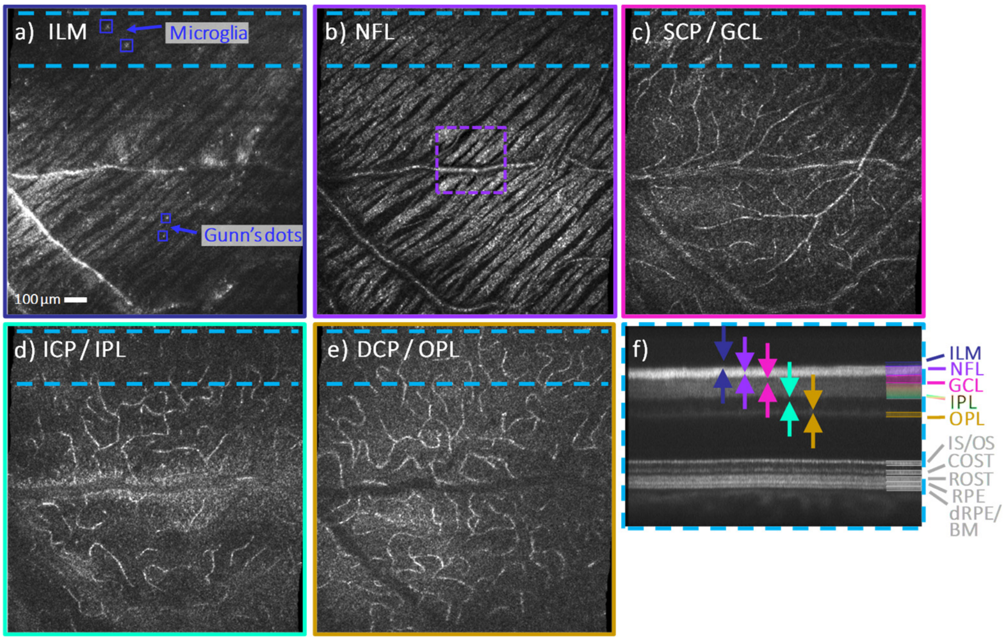

Fig. S4. Visualization of inner retinal layers using AO-OCT with the P-WFS at a large FoV of 3.81° x 3.92°. The imaging location 14° temporal / 6° superior in a healthy volunteer (29 years, male, right eye) and the focus was set to the nerve fiber layer. The representative en-face images a)-e) are extracted from a single data volume by depth integration in the inner retina over parts of or in vicinity of the following retinal layers: a) inner limiting membrane (ILM), b) nerve fiber layer (NFL) with the averaged, small FoV image of Fig. 7b) as inset, c) superficial capillary plexus (SCP) in Ganglion cell layer (GCL), d) intermediate capillary plexus (ICP) in outer part of inner plexiform layer (IPL), e) deep capillary plexus (DCP) in outer plexiform layer (OPL). The integration ranges are indicated by the color-coded arrows in the averaged B-scan f) which consists of a projection of part of the volume along the slow imaging axis which is indicated by the dashed lines in the en-face images.

In **Fig. S4**, the large FoV AO-OCT imaging data of the inner retina are displayed. In the averaged B-scan of **Fig. S4f**), the successful focus shift can be observed in the increased signal strength received from the inner retinal layers, especially the NFL. Lateral features of the inner retina can be observed at a high resolution over a larger imaging area in the en-face visualizations: The sparse distribution of the highly reflective circular and irregular structures providing evidence of Gunn's dots [7] and microglia [8] in the ILM (**Fig. S4a**) and the complex web of nerve fiber bundles of varying size dispersing across the NFL (**Fig. S4b**). The en-face slices extracted at the GCL (**Fig. S4c**), at the outer most part of the IPL (**Fig. S4d**) and at the

bright band allocated with the OPL (**Fig. S4e**) show vessel patterns of the superficial, intermediate and deep capillary plexus, respectively. The areas between the vessels show patterns and irregularities, but the signal-to-noise ratio in the single-shot volumes is not sufficient to visualize cellular structures. The large pupil size of the subject leads to a small depth of focus and the appearance of the intermediate and deep capillary plexus is slightly blurred. Due to the high numerical aperture provided by AO OCT, the vessel pattern appears with good contrast without the use of OCT angiography [9].

References

1. M. Laslandes, M. Salas, C. K. Hitzenberger, and M. Pircher, "Influence of wave-front sampling in adaptive optics retinal imaging," Biomed. Opt. Express **8 (2)**, 1083-1100 (2017).
2. Z. L. Liu, O. P. Kocaoglu, and D. T. Miller, "3D Imaging of Retinal Pigment Epithelial Cells in the Living Human Retina," Invest. Ophth. Vis. Sci. **57 (9)**, Oct533-Oct543 (2016).
3. M. F. Shirazi, E. Brunner, M. Laslandes, A. Pollreisz, C. K. Hitzenberger, and M. Pircher, "Visualizing human photoreceptor and retinal pigment epithelium cell mosaics in a single volume scan over an extended field of view with adaptive optics optical coherence tomography," Biomed. Opt. Express **11 (8)**, 4520-4535 (2020).
4. C. A. Curcio, K. R. Sloan, R. E. Kalina, and A. E. Hendrickson, "Human Photoreceptor Topography," J. Comp. Neurol. **292 (4)**, 497-523 (1990).
5. F. Felberer, J. S. Kroisamer, B. Baumann, S. Zotter, U. Schmidt-Erfurth, C. K. Hitzenberger, and M. Pircher, "Adaptive optics SLO/OCT for 3D imaging of human photoreceptors in vivo," Biomed. Opt. Express **5 (2)**, 439-456 (2014).
6. M. Pircher, J. S. Kroisamer, F. Felberer, H. Sattmann, E. Gotzinger, and C. K. Hitzenberger, "Temporal changes of human cone photoreceptors observed in vivo with SLO/OCT," Biomed. Opt. Express **2 (1)**, 100-112 (2011).
7. D. X. Hammer, Z. Liu, J. A. Cava, J. Carroll, and O. Saeedi, "On the axial location of Gunn's dots," Am. J. Ophthalmol. Case. Rep. **19**, 100757 (2020).
8. Z. L. Liu, J. Tam, O. Saeedi, and D. X. Hammer, "Trans-retinal cellular imaging with multimodal adaptive optics," Biomed. Opt. Express **9 (9)**, 4246-4262 (2018).
9. M. Salas, M. Augustin, L. Ginner, A. Kumar, B. Baumann, R. Leitgeb, W. Drexler, S. Prager, J. Hafner, U. Schmidt-Erfurth, and M. Pircher, "Visualization of micro-capillaries using optical coherence tomography angiography with and without adaptive optics," Biomed. Opt. Express **8 (1)**, 207-222 (2017).
